# 4-(1*H*-Benzimidazol-2-ylmeth­yl)-2*H*-1,4-benzothia­zin-3(4*H*)-one

**DOI:** 10.1107/S1600536810019367

**Published:** 2010-05-29

**Authors:** Hoong-Kun Fun, Mohd Mustaqim Rosli, Janardhana Gowda, A. M. A. Khader, B. Kalluraya

**Affiliations:** a X-ray Crystallography Unit, School of Physics, Universiti Sains Malaysia, 11800 USM, Penang, Malaysia; b Department of Studies in Chemistry, Mangalore University, Mangalagangotri, Mangalore 574 199, India

## Abstract

In the title compound, C_16_H_13_N_3_OS, the thio­morpholine ring exists in a screw boat conformation. The angle between the benzimidazole ring system and the benzene ring fused to the thia­zine ring is 67.22 (6)°. In the crystal, mol­ecules form infinite chains along the *a* axis *via* inter­molecular N—H⋯N inter­actions. C—H⋯π inter­actions also contribute to the stability of the crystal structure.

## Related literature

For the biological activity of mol­ecules containing 1*H*-benzimidazole, see: Sridhar & Ramesh (2001 ▶); Guven *et al.* (2007 ▶); Nofal *et al.* (2002 ▶); Pedini *et al.* (1994 ▶). For a related structure, see: Fun *et al.* (2009 ▶). For ring puckering parameters, see: Cremer & Pople (1975 ▶). For the stability of the temperature controller used for the data collection, see: Cosier & Glazer (1986 ▶).
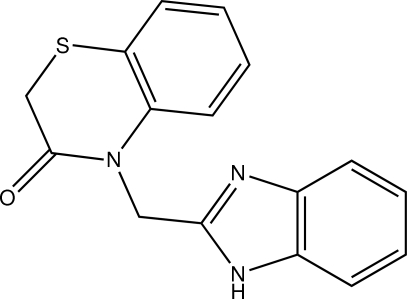


## Experimental

### 

#### Crystal data

C_16_H_13_N_3_OS
*M* *_r_* = 295.35
Orthorhombic, 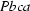

*a* = 9.4498 (8) Å
*b* = 17.0223 (16) Å
*c* = 17.4454 (16) Å
*V* = 2806.2 (4) Å^3^
*Z* = 8
Mo *K*α radiation
μ = 0.23 mm^−1^
*T* = 100 K
0.50 × 0.20 × 0.13 mm

#### Data collection

Bruker APEXII DUO CCD area-detector diffractometer
Absorption correction: multi-scan (*SADABS*; Bruker, 2009 ▶) *T*_min_ = 0.893, *T*_max_ = 0.969
16727 measured reflections
4075 independent reflections
3185 reflections with *I* > 2σ(*I*)
*R*_int_ = 0.040

#### Refinement

*R*[*F* ^2^ > 2σ(*F* ^2^)] = 0.041
*wR*(*F* ^2^) = 0.119
*S* = 1.03
4075 reflections
194 parameters
H atoms treated by a mixture of independent and constrained refinement
Δρ_max_ = 0.44 e Å^−3^
Δρ_min_ = −0.26 e Å^−3^

### 

Data collection: *APEX2* (Bruker, 2009 ▶); cell refinement: *SAINT* (Bruker, 2009 ▶); data reduction: *SAINT*; program(s) used to solve structure: *SHELXTL* (Sheldrick, 2008 ▶); program(s) used to refine structure: *SHELXTL*; molecular graphics: *SHELXTL*; software used to prepare material for publication: *SHELXTL* and *PLATON* (Spek, 2009 ▶).

## Supplementary Material

Crystal structure: contains datablocks global, I. DOI: 10.1107/S1600536810019367/wn2387sup1.cif

Structure factors: contains datablocks I. DOI: 10.1107/S1600536810019367/wn2387Isup2.hkl

Additional supplementary materials:  crystallographic information; 3D view; checkCIF report

## Figures and Tables

**Table 1:** Hydrogen-bond geometry (Å, °) *Cg*1 and *Cg*2 are the centroids of the C1–C6 and C11–C16 rings, respectively.

*D*—H⋯*A*	*D*—H	H⋯*A*	*D*⋯*A*	*D*—H⋯*A*
N1—H1*N*1⋯N2^i^	0.859 (19)	1.926 (19)	2.7800 (15)	173 (2)
C12—H12*A*⋯*Cg*1^ii^	0.93	2.97	3.6736 (16)	134
C3—H3*A*⋯*Cg*2^iii^	0.93	2.61	3.4750 (17)	155

Symmetry codes: (i) 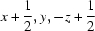; (ii) 
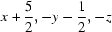
; (iii) 
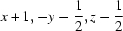
.

**Fig. 1.**
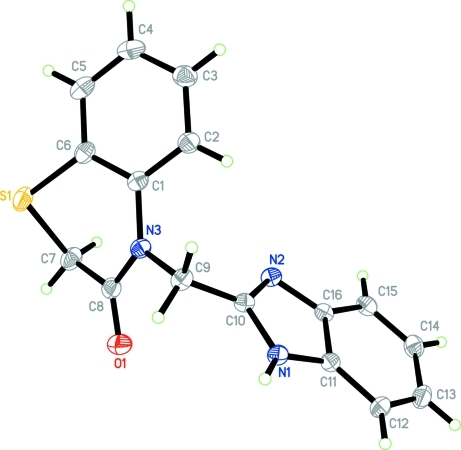
The molecular structure, showing 50% probability displacement ellipsoids and the atom-numbering scheme. Hydrogen atoms are shown as spheres of arbitrary radius.

**Fig. 2.**
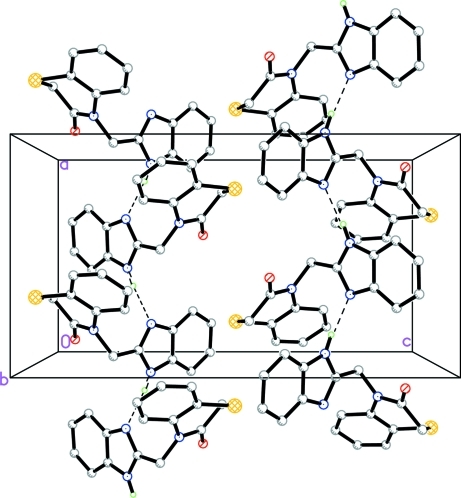
The crystal structure, showing infinite chains along the a-axis. Dashed lines indicate hydrogen bonds. H atoms not involved in the hydrogen bond interactions have been omitted for clarity.

## supplementary crystallographic information

### Comment

A number of molecules containing the 1H-benzimidazole nucleus exhibit a broad spectrum of biological activity, including anti-inflammatory (Sridhar *et al.,* 2001), antifungal (Guven *et al.,* 2007), antibacterial (Nofal *et al.,* 2002) and anthelmintic (Pedini *et al.,* 1994) properties. With these results in mind, we have paid particular attention to the preparation of derivatives of 1H-benzimidazole and we report here the crystal structure of the title compound, a 1H-benzimidazole derivative containing 2H-1,4-benzothiazin-3(4H)-one.

The bond lengths and angles are within normal ranges. The thiomorpholine ring (C1, C6-C8, N3, S1) adopts a screw boat confirmation with puckering parameters (Cremer & Pople, 1975) being Q = 0.6563 (13) Å; θ = 66.76 (12)° and φ = 334.16 (14)°. The angle between the benzimidazole ring system and the benzene ring fused to the thiazine ring is 67.22 (6)°.

The intermolecular interaction  N1—H1N1···N2 links the molecules to form infinite chains along the a-axis. The crystal structure is further stabilized by C—H···π interactions involving the C1-C6 (Cg1) and C11-C16 (Cg2) benzene rings (Table 1).

### Experimental

A mixture of 2-(3-oxo-2,3-dihydro-4H-1,4-benzothiazin-4-yl)acetic acid (3.3 mmol) (Fun *et al.,* 2009) and o-phenylenediamine (2.2 mmol) was heated at 140 °C under solvent-free conditions for 3 h and completion of the reaction was checked by TLC. The reaction mixture was cooled to room temperature and the solid product was washed with a saturated solution of sodium bicarbonate to yield 4-(1H-benzimidazol-2-ylmethyl)-2H-1,4-benzothiazin-3(4H)-one as a red solid. Single crystals suitable for X-ray analysis were obtained by crystallization from absolute ethanol under slow evaporation (M.p. 493 K).

### Refinement

The H atom attached to N1 was located in a difference map and refined isotropically; N1—H1N1 =  0.86 (2) Å. The carbon-bound H atoms were positioned geometrically [C—H = 0.93 or 0.97 Å] and were refined using a riding model, with U_iso_(H) = 1.2U_eq_(C).

### Crystal data

**Table:**

C_16_H_13_N_3_OS	*F*(000) = 1232
*M**_r_* = 295.35	*D*_x_ = 1.398 Mg m^−^^3^
Orthorhombic, *P**b**c**a*	Mo *K*α radiation, λ = 0.71073 Å
Hall symbol: -P 2ac 2ab	Cell parameters from 4703 reflections
*a* = 9.4498 (8) Å	θ = 2.4–31.6°
*b* = 17.0223 (16) Å	µ = 0.23 mm^−^^1^
*c* = 17.4454 (16) Å	*T* = 100 K
*V* = 2806.2 (4) Å^3^	Block, red
*Z* = 8	0.50 × 0.20 × 0.13 mm

### Data collection

**Table:**

Bruker APEXII DUO CCD area-detector diffractometer	4075 independent reflections
Radiation source: fine-focus sealed tube	3185 reflections with *I* > 2σ(*I*)
graphite	*R*_int_ = 0.040
φ and ω scans	θ_max_ = 30.0°, θ_min_ = 2.3°
Absorption correction: multi-scan (*SADABS*; Bruker, 2009)	*h* = −13→13
*T*_min_ = 0.893, *T*_max_ = 0.969	*k* = −23→23
16727 measured reflections	*l* = −24→21

### Refinement

**Table:**

Refinement on *F*^2^	Primary atom site location: structure-invariant direct methods
Least-squares matrix: full	Secondary atom site location: difference Fourier map
*R*[*F*^2^ > 2σ(*F*^2^)] = 0.041	Hydrogen site location: inferred from neighbouring sites
*wR*(*F*^2^) = 0.119	H atoms treated by a mixture of independent and constrained refinement
*S* = 1.03	*w* = 1/[σ^2^(*F*_o_^2^) + (0.0605*P*)^2^ + 1.2202*P*] where *P* = (*F*_o_^2^ + 2*F*_c_^2^)/3
4075 reflections	(Δ/σ)_max_ = 0.001
194 parameters	Δρ_max_ = 0.44 e Å^−^^3^
0 restraints	Δρ_min_ = −0.25 e Å^−^^3^

### Special details

**Table:**

Experimental. The crystal was placed in the cold stream of an Oxford Cyrosystems Cobra open-flow nitrogen cryostat (Cosier & Glazer, 1986) operating at 100.0 (1) K.
Geometry. All esds (except the esd in the dihedral angle between two l.s. planes) are estimated using the full covariance matrix. The cell esds are taken into account individually in the estimation of esds in distances, angles and torsion angles; correlations between esds in cell parameters are only used when they are defined by crystal symmetry. An approximate (isotropic) treatment of cell esds is used for estimating esds involving l.s. planes.
Refinement. Refinement of F^2^ against ALL reflections. The weighted R-factor wR and goodness of fit S are based on F^2^, conventional R-factors R are based on F, with F set to zero for negative F^2^. The threshold expression of F^2^ > 2sigma(F^2^) is used only for calculating R-factors(gt) etc. and is not relevant to the choice of reflections for refinement. R-factors based on F^2^ are statistically about twice as large as those based on F, and R- factors based on ALL data will be even larger.

### Fractional atomic coordinates and isotropic or equivalent isotropic displacement parameters (Å^2^)

**Table:**

	*x*	*y*	*z*	*U*_iso_*/*U*_eq_	
S1	0.69098 (4)	0.04490 (2)	0.49857 (2)	0.02567 (12)	
O1	0.90053 (12)	−0.12536 (7)	0.41611 (7)	0.0283 (2)	
N1	1.05774 (11)	−0.12424 (7)	0.21967 (7)	0.0169 (2)	
N2	0.82471 (11)	−0.10176 (7)	0.21708 (7)	0.0166 (2)	
N3	0.84628 (12)	−0.01064 (7)	0.35843 (7)	0.0180 (2)	
C1	0.75457 (14)	0.05425 (8)	0.34596 (8)	0.0177 (3)	
C2	0.74342 (15)	0.08809 (8)	0.27346 (8)	0.0210 (3)	
H2A	0.7935	0.0668	0.2325	0.025*	
C3	0.65796 (16)	0.15354 (9)	0.26189 (9)	0.0251 (3)	
H3A	0.6530	0.1764	0.2135	0.030*	
C4	0.58032 (16)	0.18488 (9)	0.32169 (10)	0.0275 (3)	
H4A	0.5232	0.2286	0.3137	0.033*	
C5	0.58816 (16)	0.15078 (9)	0.39359 (10)	0.0256 (3)	
H5A	0.5346	0.1711	0.4337	0.031*	
C6	0.67593 (15)	0.08616 (8)	0.40635 (8)	0.0206 (3)	
C7	0.70051 (17)	−0.05535 (9)	0.46596 (9)	0.0261 (3)	
H7A	0.7100	−0.0900	0.5098	0.031*	
H7B	0.6134	−0.0688	0.4396	0.031*	
C8	0.82425 (15)	−0.06773 (8)	0.41241 (8)	0.0209 (3)	
C9	0.96991 (14)	−0.02207 (8)	0.30913 (8)	0.0191 (3)	
H9A	1.0499	−0.0374	0.3406	0.023*	
H9B	0.9933	0.0274	0.2847	0.023*	
C10	0.94729 (13)	−0.08304 (8)	0.24863 (8)	0.0159 (2)	
C11	1.00375 (13)	−0.17492 (8)	0.16502 (8)	0.0165 (3)	
C12	1.06703 (15)	−0.23109 (9)	0.11796 (8)	0.0213 (3)	
H12A	1.1636	−0.2415	0.1201	0.026*	
C13	0.97847 (17)	−0.27045 (9)	0.06787 (9)	0.0245 (3)	
H13A	1.0162	−0.3086	0.0356	0.029*	
C14	0.83220 (16)	−0.25414 (9)	0.06449 (9)	0.0234 (3)	
H14A	0.7765	−0.2809	0.0291	0.028*	
C15	0.76917 (15)	−0.19954 (8)	0.11228 (8)	0.0198 (3)	
H15A	0.6724	−0.1898	0.1105	0.024*	
C16	0.85770 (13)	−0.15965 (8)	0.16350 (8)	0.0158 (2)	
H1N1	1.143 (2)	−0.1173 (12)	0.2353 (12)	0.033 (5)*	

### Atomic displacement parameters (Å^2^)

**Table:**

	*U*^11^	*U*^22^	*U*^33^	*U*^12^	*U*^13^	*U*^23^
S1	0.0341 (2)	0.02221 (19)	0.02070 (19)	−0.00054 (14)	0.00590 (14)	−0.00461 (13)
O1	0.0333 (6)	0.0235 (5)	0.0283 (6)	0.0070 (4)	0.0003 (5)	0.0023 (4)
N1	0.0100 (5)	0.0214 (6)	0.0192 (5)	−0.0003 (4)	−0.0003 (4)	−0.0010 (4)
N2	0.0117 (5)	0.0174 (5)	0.0208 (6)	−0.0010 (4)	0.0004 (4)	−0.0003 (4)
N3	0.0166 (5)	0.0180 (5)	0.0194 (6)	0.0007 (4)	0.0020 (4)	−0.0006 (4)
C1	0.0157 (6)	0.0154 (6)	0.0221 (6)	−0.0018 (4)	0.0004 (5)	−0.0017 (5)
C2	0.0220 (6)	0.0180 (6)	0.0230 (7)	−0.0014 (5)	0.0003 (5)	−0.0005 (5)
C3	0.0282 (7)	0.0192 (7)	0.0279 (8)	−0.0014 (5)	−0.0046 (6)	0.0018 (6)
C4	0.0238 (7)	0.0185 (6)	0.0402 (9)	0.0032 (5)	−0.0036 (6)	−0.0013 (6)
C5	0.0220 (7)	0.0214 (7)	0.0334 (8)	0.0014 (5)	0.0045 (6)	−0.0069 (6)
C6	0.0201 (6)	0.0187 (6)	0.0230 (7)	−0.0025 (5)	0.0021 (5)	−0.0039 (5)
C7	0.0349 (8)	0.0197 (7)	0.0236 (7)	−0.0006 (6)	0.0088 (6)	−0.0004 (6)
C8	0.0247 (7)	0.0187 (6)	0.0192 (7)	−0.0005 (5)	−0.0001 (5)	−0.0014 (5)
C9	0.0133 (5)	0.0209 (6)	0.0232 (7)	−0.0019 (5)	0.0004 (5)	−0.0036 (5)
C10	0.0116 (5)	0.0173 (6)	0.0188 (6)	−0.0007 (4)	0.0018 (5)	0.0013 (5)
C11	0.0138 (5)	0.0191 (6)	0.0167 (6)	−0.0002 (4)	0.0006 (5)	0.0011 (5)
C12	0.0182 (6)	0.0245 (7)	0.0211 (7)	0.0030 (5)	0.0035 (5)	−0.0009 (5)
C13	0.0293 (7)	0.0238 (7)	0.0204 (7)	0.0013 (6)	0.0038 (6)	−0.0042 (5)
C14	0.0256 (7)	0.0241 (7)	0.0204 (7)	−0.0036 (5)	−0.0026 (5)	−0.0012 (5)
C15	0.0171 (6)	0.0214 (6)	0.0211 (7)	−0.0029 (5)	−0.0027 (5)	0.0019 (5)
C16	0.0134 (5)	0.0171 (6)	0.0168 (6)	−0.0011 (4)	0.0003 (5)	0.0030 (5)

### Geometric parameters (Å, °)

**Table:**

S1—C6	1.7611 (16)	C5—C6	1.396 (2)
S1—C7	1.8011 (16)	C5—H5A	0.9300
O1—C8	1.2191 (18)	C7—C8	1.511 (2)
N1—C10	1.3552 (16)	C7—H7A	0.9700
N1—C11	1.3831 (17)	C7—H7B	0.9700
N1—H1N1	0.86 (2)	C9—C10	1.4955 (19)
N2—C10	1.3214 (16)	C9—H9A	0.9700
N2—C16	1.3936 (17)	C9—H9B	0.9700
N3—C8	1.3692 (19)	C11—C12	1.3950 (19)
N3—C1	1.4207 (17)	C11—C16	1.4046 (18)
N3—C9	1.4637 (17)	C12—C13	1.383 (2)
C1—C2	1.394 (2)	C12—H12A	0.9300
C1—C6	1.3990 (19)	C13—C14	1.411 (2)
C2—C3	1.391 (2)	C13—H13A	0.9300
C2—H2A	0.9300	C14—C15	1.383 (2)
C3—C4	1.382 (2)	C14—H14A	0.9300
C3—H3A	0.9300	C15—C16	1.3998 (19)
C4—C5	1.384 (2)	C15—H15A	0.9300
C4—H4A	0.9300		
			
C6—S1—C7	95.35 (7)	H7A—C7—H7B	108.0
C10—N1—C11	107.20 (11)	O1—C8—N3	121.18 (13)
C10—N1—H1N1	122.3 (14)	O1—C8—C7	122.47 (14)
C11—N1—H1N1	130.5 (14)	N3—C8—C7	116.35 (12)
C10—N2—C16	104.70 (11)	N3—C9—C10	113.14 (11)
C8—N3—C1	124.36 (12)	N3—C9—H9A	109.0
C8—N3—C9	115.55 (12)	C10—C9—H9A	109.0
C1—N3—C9	120.02 (11)	N3—C9—H9B	109.0
C2—C1—C6	118.86 (13)	C10—C9—H9B	109.0
C2—C1—N3	120.42 (12)	H9A—C9—H9B	107.8
C6—C1—N3	120.71 (13)	N2—C10—N1	113.27 (12)
C3—C2—C1	120.45 (14)	N2—C10—C9	125.91 (12)
C3—C2—H2A	119.8	N1—C10—C9	120.81 (11)
C1—C2—H2A	119.8	N1—C11—C12	132.45 (12)
C4—C3—C2	120.52 (15)	N1—C11—C16	105.08 (11)
C4—C3—H3A	119.7	C12—C11—C16	122.47 (13)
C2—C3—H3A	119.7	C13—C12—C11	116.39 (13)
C3—C4—C5	119.58 (14)	C13—C12—H12A	121.8
C3—C4—H4A	120.2	C11—C12—H12A	121.8
C5—C4—H4A	120.2	C12—C13—C14	121.59 (13)
C4—C5—C6	120.46 (14)	C12—C13—H13A	119.2
C4—C5—H5A	119.8	C14—C13—H13A	119.2
C6—C5—H5A	119.8	C15—C14—C13	121.92 (14)
C5—C6—C1	120.11 (14)	C15—C14—H14A	119.0
C5—C6—S1	120.53 (11)	C13—C14—H14A	119.0
C1—C6—S1	119.35 (11)	C14—C15—C16	116.96 (13)
C8—C7—S1	111.46 (10)	C14—C15—H15A	121.5
C8—C7—H7A	109.3	C16—C15—H15A	121.5
S1—C7—H7A	109.3	N2—C16—C15	129.61 (12)
C8—C7—H7B	109.3	N2—C16—C11	109.75 (11)
S1—C7—H7B	109.3	C15—C16—C11	120.64 (13)
			
C8—N3—C1—C2	−150.61 (14)	C8—N3—C9—C10	76.17 (15)
C9—N3—C1—C2	25.96 (19)	C1—N3—C9—C10	−100.69 (14)
C8—N3—C1—C6	30.5 (2)	C16—N2—C10—N1	0.06 (15)
C9—N3—C1—C6	−152.88 (13)	C16—N2—C10—C9	178.82 (13)
C6—C1—C2—C3	1.4 (2)	C11—N1—C10—N2	−0.62 (16)
N3—C1—C2—C3	−177.44 (13)	C11—N1—C10—C9	−179.45 (12)
C1—C2—C3—C4	−1.5 (2)	N3—C9—C10—N2	28.7 (2)
C2—C3—C4—C5	0.1 (2)	N3—C9—C10—N1	−152.62 (12)
C3—C4—C5—C6	1.3 (2)	C10—N1—C11—C12	−179.20 (15)
C4—C5—C6—C1	−1.4 (2)	C10—N1—C11—C16	0.88 (14)
C4—C5—C6—S1	177.68 (12)	N1—C11—C12—C13	−178.56 (14)
C2—C1—C6—C5	0.0 (2)	C16—C11—C12—C13	1.3 (2)
N3—C1—C6—C5	178.85 (13)	C11—C12—C13—C14	0.3 (2)
C2—C1—C6—S1	−179.06 (10)	C12—C13—C14—C15	−1.7 (2)
N3—C1—C6—S1	−0.20 (18)	C13—C14—C15—C16	1.3 (2)
C7—S1—C6—C5	142.33 (13)	C10—N2—C16—C15	−178.42 (14)
C7—S1—C6—C1	−38.62 (13)	C10—N2—C16—C11	0.52 (15)
C6—S1—C7—C8	58.75 (12)	C14—C15—C16—N2	179.23 (13)
C1—N3—C8—O1	173.88 (13)	C14—C15—C16—C11	0.4 (2)
C9—N3—C8—O1	−2.8 (2)	N1—C11—C16—N2	−0.88 (15)
C1—N3—C8—C7	−5.6 (2)	C12—C11—C16—N2	179.20 (12)
C9—N3—C8—C7	177.65 (12)	N1—C11—C16—C15	178.18 (12)
S1—C7—C8—O1	137.50 (14)	C12—C11—C16—C15	−1.8 (2)
S1—C7—C8—N3	−42.98 (17)		

### Hydrogen-bond geometry (Å, °)

**Table:**

Cg1 and Cg2 are the centroids of the C1–C6 and C11–C16 rings, respectively.

**Table:**

*D*—H···*A*	*D*—H	H···*A*	*D*···*A*	*D*—H···*A*
N1—H1N1···N2^i^	0.859 (19)	1.926 (19)	2.7800 (15)	173 (2)
C12—H12A···Cg1^ii^	0.93	2.97	3.6736 (16)	134
C3—H3A···Cg2^iii^	0.93	2.61	3.4750 (17)	155

Symmetry codes: (i) *x*+1/2, *y*, −*z*+1/2; (ii) *x*+5/2, −*y*−1/2, −*z*; (iii) *x*+1, −*y*−1/2, *z*−1/2.
